# Supplementary material for: Mass Cytometric Analysis of Early-Stage Mycosis Fungoides
Source: Cells. 2022 Mar 22;11(7):1062. doi: 10.3390/cells11071062 (PMC8997708; doi:10.3390/cells11071062)
Supplement: Supplementary file 1 [file cells-11-01062-s001.zip › cells-1600054-supplementary.pdf]

Supplementary Information

Figure S1 Gating strategy for single, live CD45$^+$ cells on MF samples (A), and identification of the overview level of CD45$^+$ cells by HSNE analysis (B).

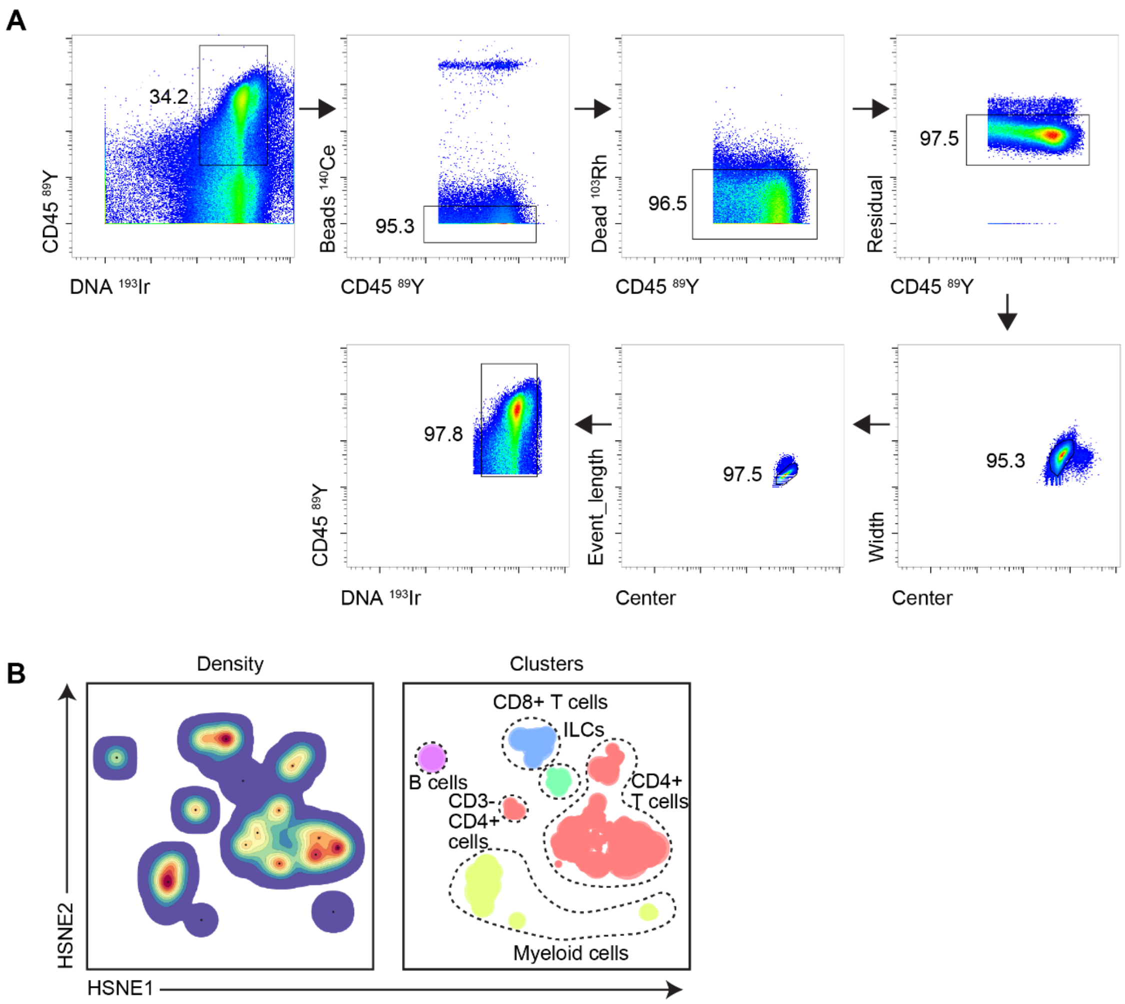

Figure S2 A collective t-SNE was performed on $CD3^{-}CD4^{+}$ T cells, and show the density map showing the local probability density of the embedded cells (left); Colors represent cluster partitions for per $CD4^{+}$ T cells subpopulation (right).

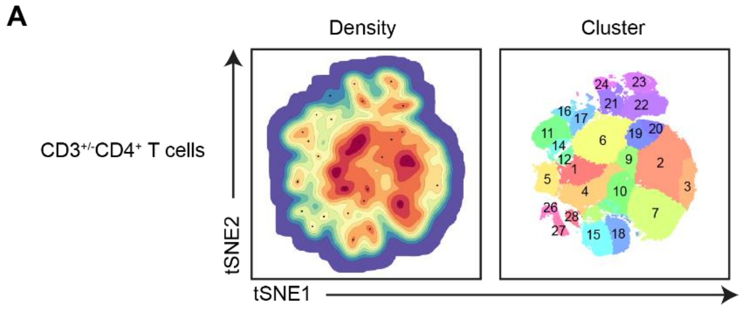

Figure S3 Identification of phenotypically distinct clusters in the $CD3^{+}CD8^{+}$ T cell compartment across MF samples. **(A)** A collective t-SNE was performed on $CD8^{+}$ T cells and stratified for samples (n = 10). In total, 5.1×10^{4} $CD8^{+}$ T cells were analyzed in the plots. **(B)** Relative expression level of indicated immune markers. Colors represent different level of marker expression. **(C)** The density map showing the local probability density of the embedded cells (left); Colors represent cluster partitions for per $CD8^{+}$ T cells subpopulation (right). **(D)** Heatmap (blue-to-red scale) showing the median of marker expression values for the identified clusters and hierarchical clustering thereof; heatmap (green-to-yellow scale) showing the corresponding cell frequencies of identified clusters of total $CD8^{+}$ T cells in each sample. The dendrogram shows the hierarchical clustering of samples. Colors represent different samples.

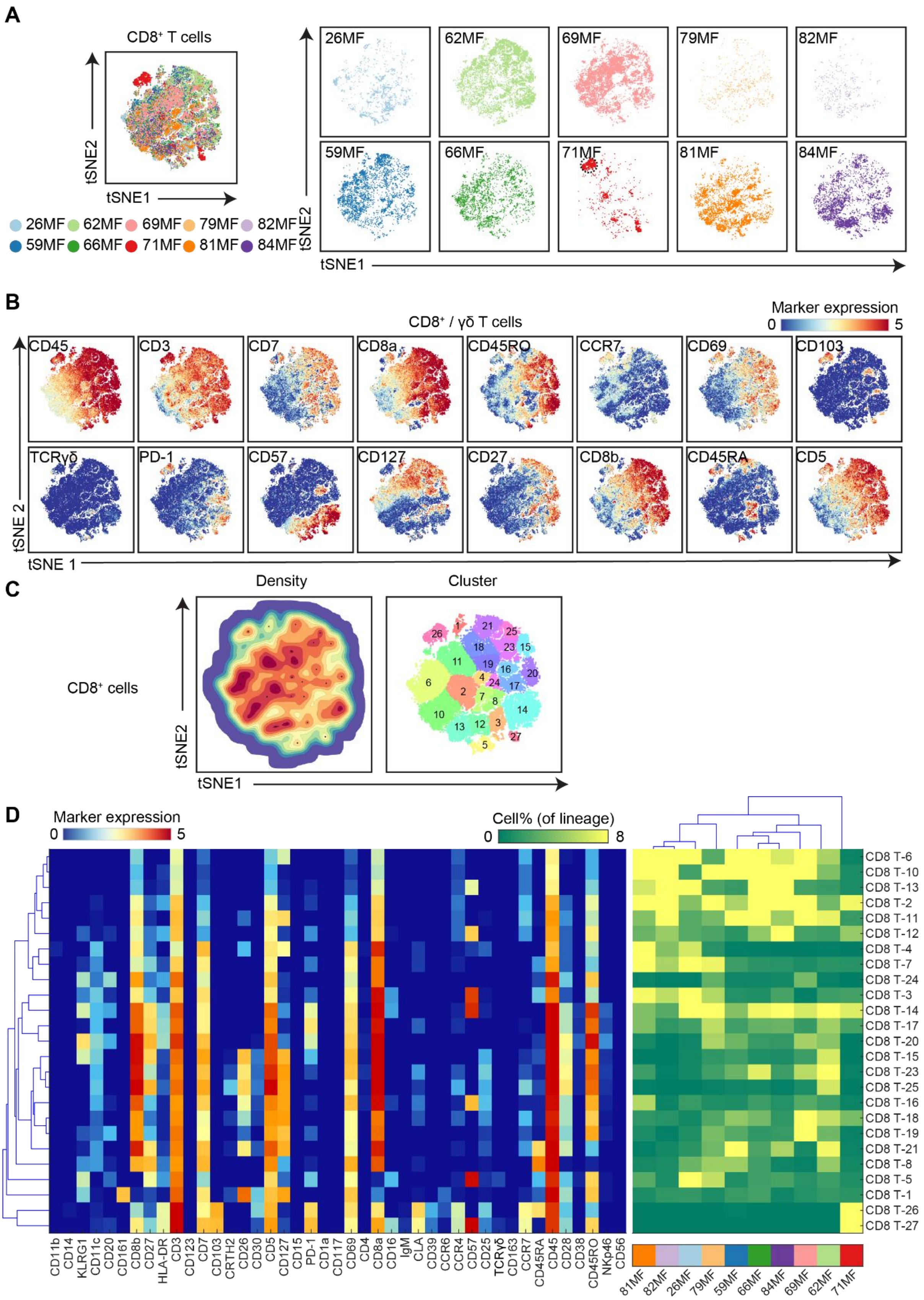

A
CD8⁺ T cells
tSNE1
tSNE2
26MF 62MF 69MF 79MF 82MF
59MF 66MF 71MF 81MF 84MF
B
CD8⁺ / γδ T cells
Marker expression
0 5
CD45 CD3 CD7 CD8a CD45RO CCR7 CD69 CD103
TCRγδ PD-1 CD57 CD127 CD27 CD8b CD45RA CD5
tSNE 1
tSNE 2
C
Density
Cluster
CD8⁺ cells
D
Marker expression
Cell% (of lineage)
CD8 T-6
CD8 T-10
CD8 T-13
CD8 T-2
CD8 T-11
CD8 T-12
CD8 T-4
CD8 T-7
CD8 T-24
CD8 T-3
CD8 T-14
CD8 T-17
CD8 T-20
CD8 T-15
CD8 T-23
CD8 T-25
CD8 T-16
CD8 T-18
CD8 T-19
CD8 T-21
CD8 T-8
CD8 T-5
CD8 T-1
CD8 T-26
CD8 T-27
CD11b CD14 KLRG1 CD11c CD20 CD161 CD8b CD27 HLA-DR CD3 CD123 CD7 CD103 CRTH2 CD26 CD30 CD5 CD127 CD15 PD-1 CD1a CD117 CD69 CD4 CD8a CD16 IgM CLA CD39 CCR6 CCR4 CD57 CD25 TCRγδ CD163 CCR7 CD45RA CD45 CD28 CD38 CD45RO NKp46 CD56
81MF 82MF 26MF 79MF 59MF 66MF 84MF 69MF 62MF 71MF

Figure S4 A collective t-SNE was performed on myeloid cells. The density map showing the local probability density of the embedded cells (left); Colors represent cluster partitions for per myeloid cells subpopulation (right).

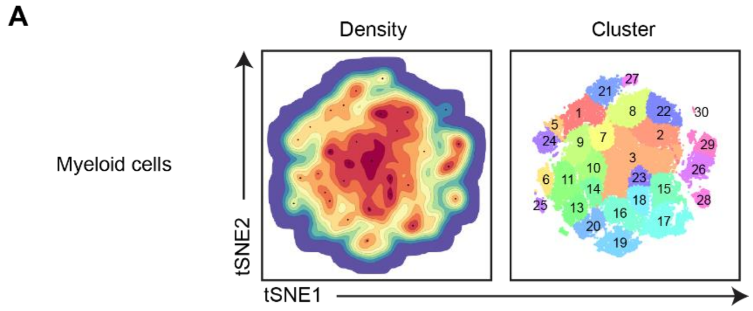

Figure S5 Individual antibody stains for representative MF patients (120MF) by IMC.

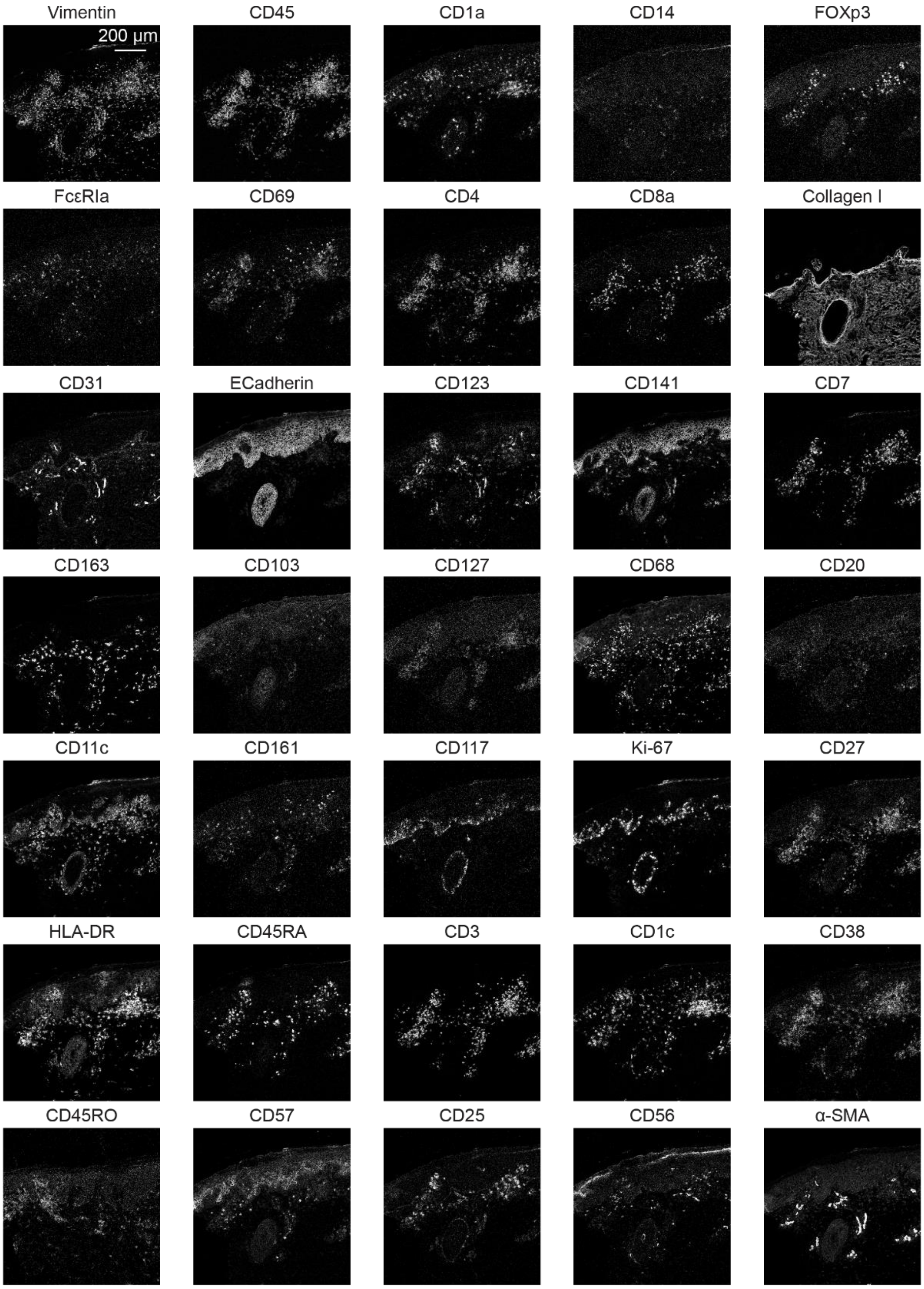

Figure S6 Individual antibody stains for 87MF patients by IMC.

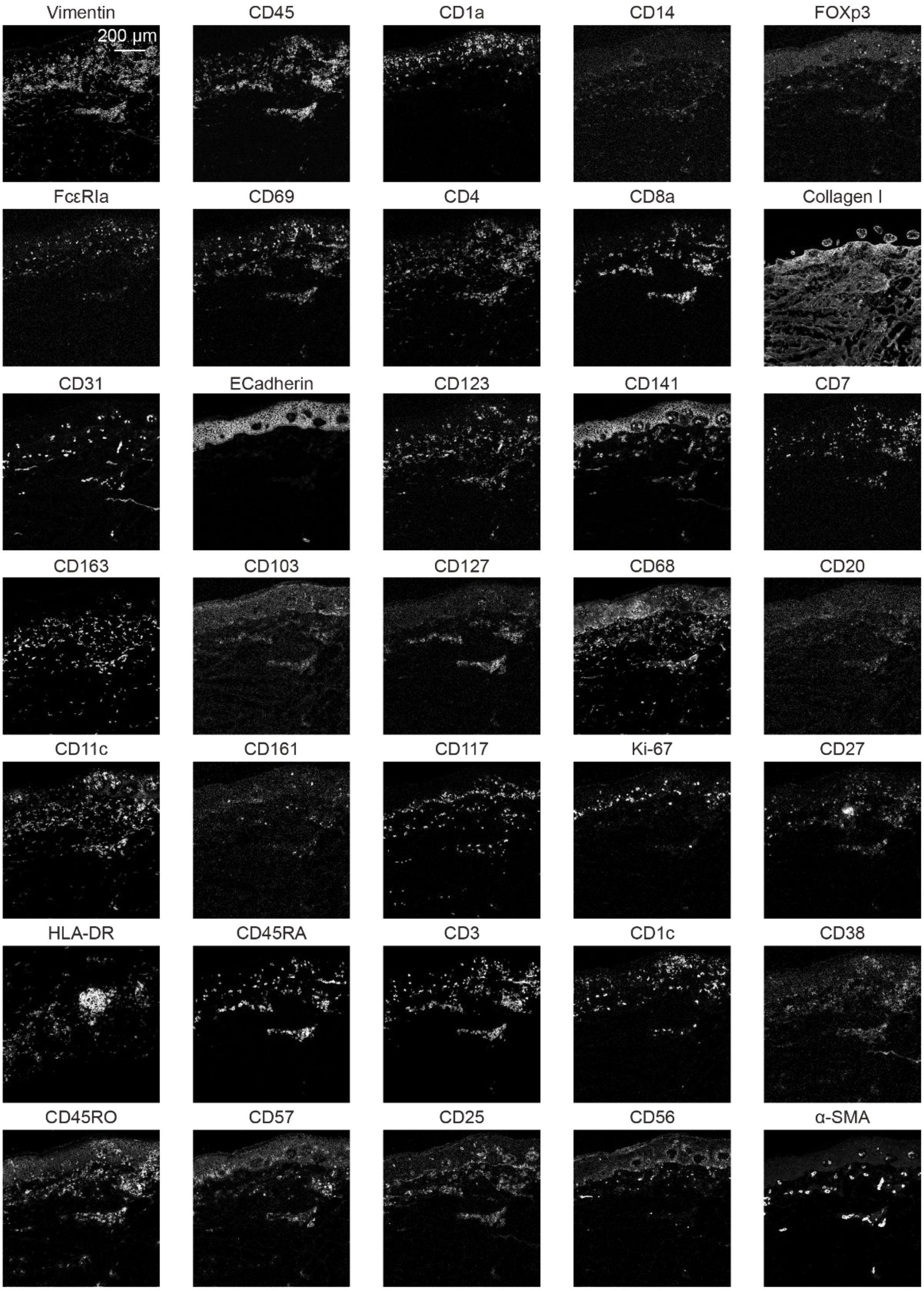

Figure S7 Individual antibody stains for 105MF patients by IMC.

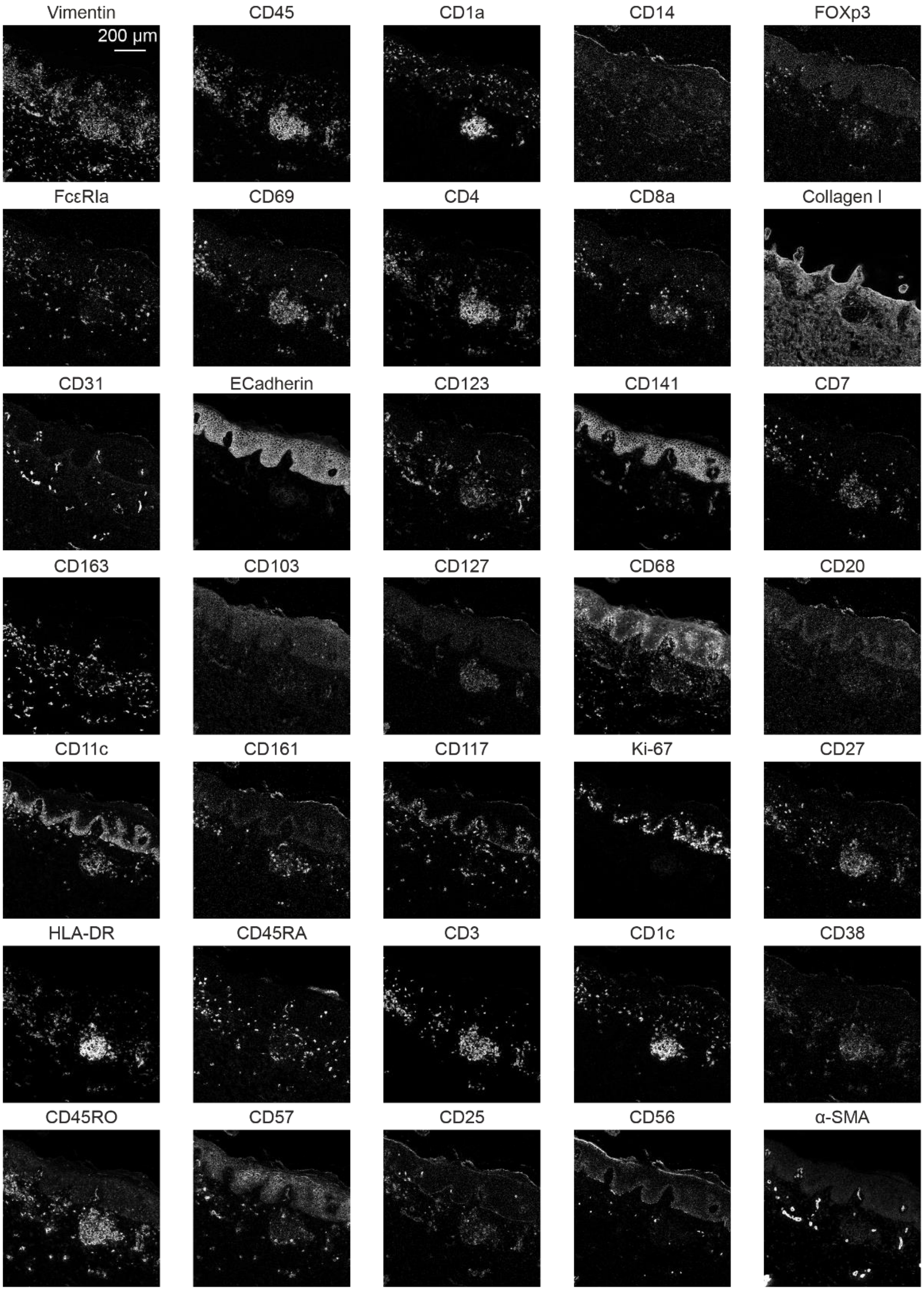

Figure S8 Individual antibody stains for 108MF patients by IMC.

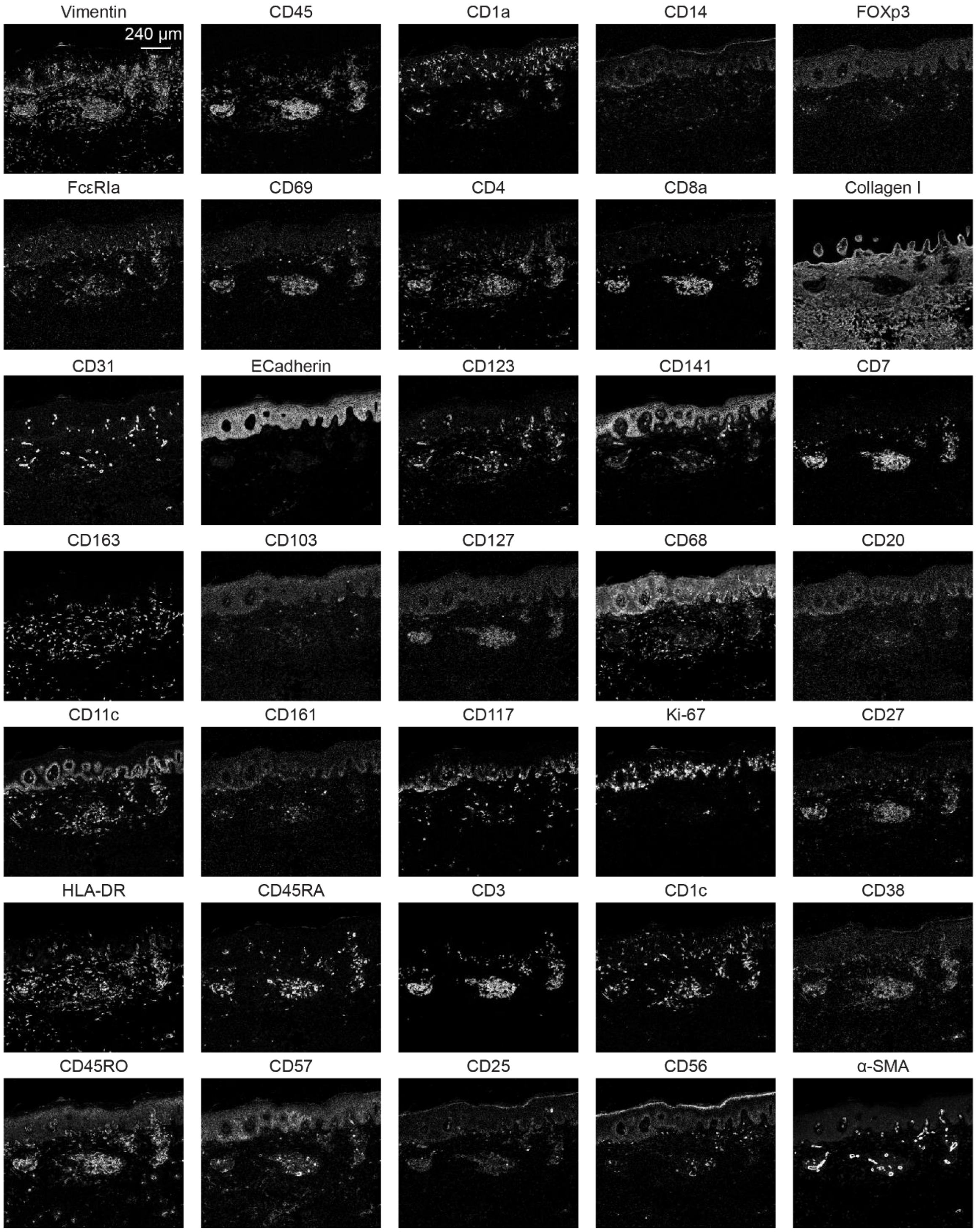

Figure S9 Individual antibody stains for 109MF patients by IMC.

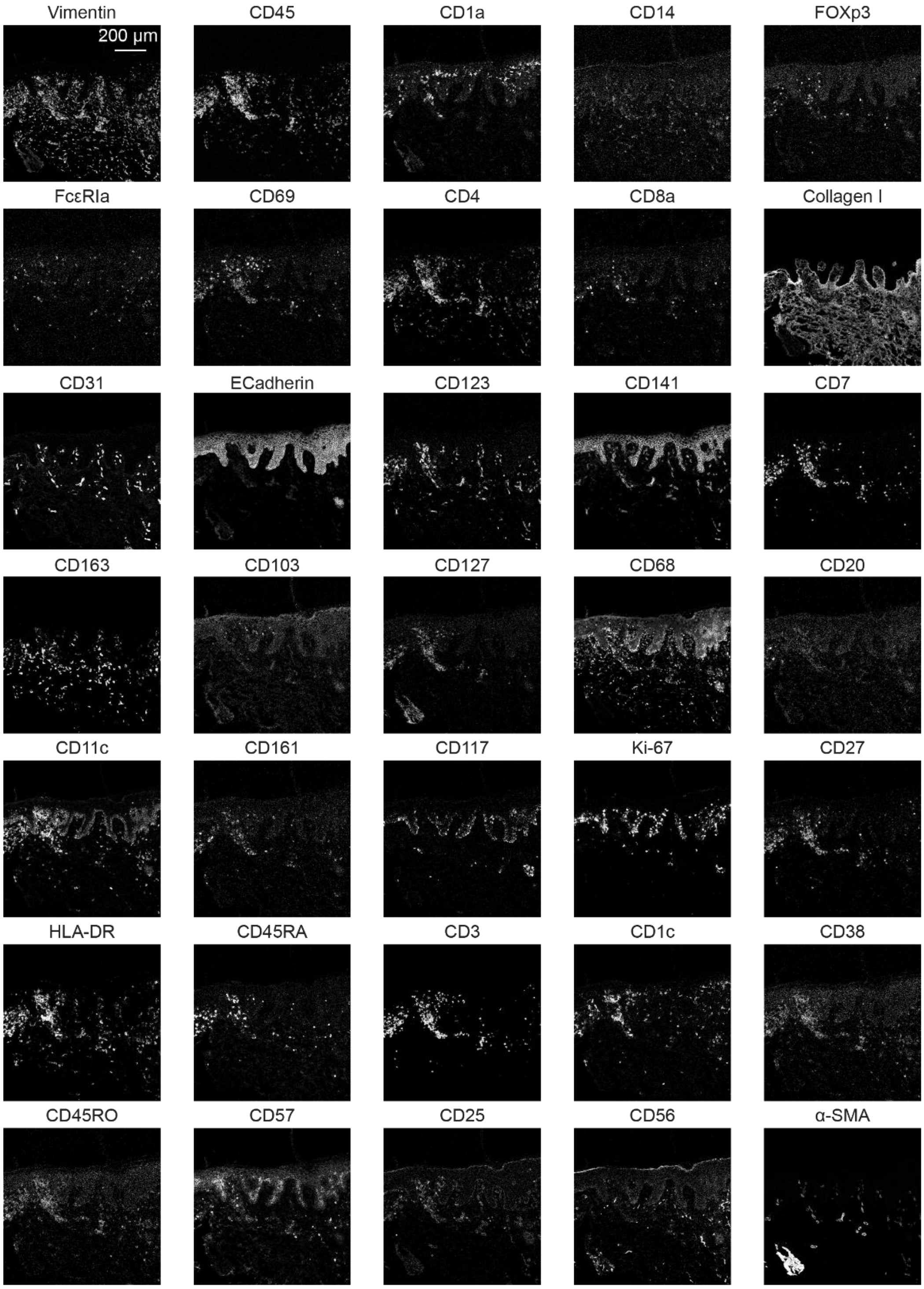

Figure S10 Individual antibody stains for 113MF patients by IMC.

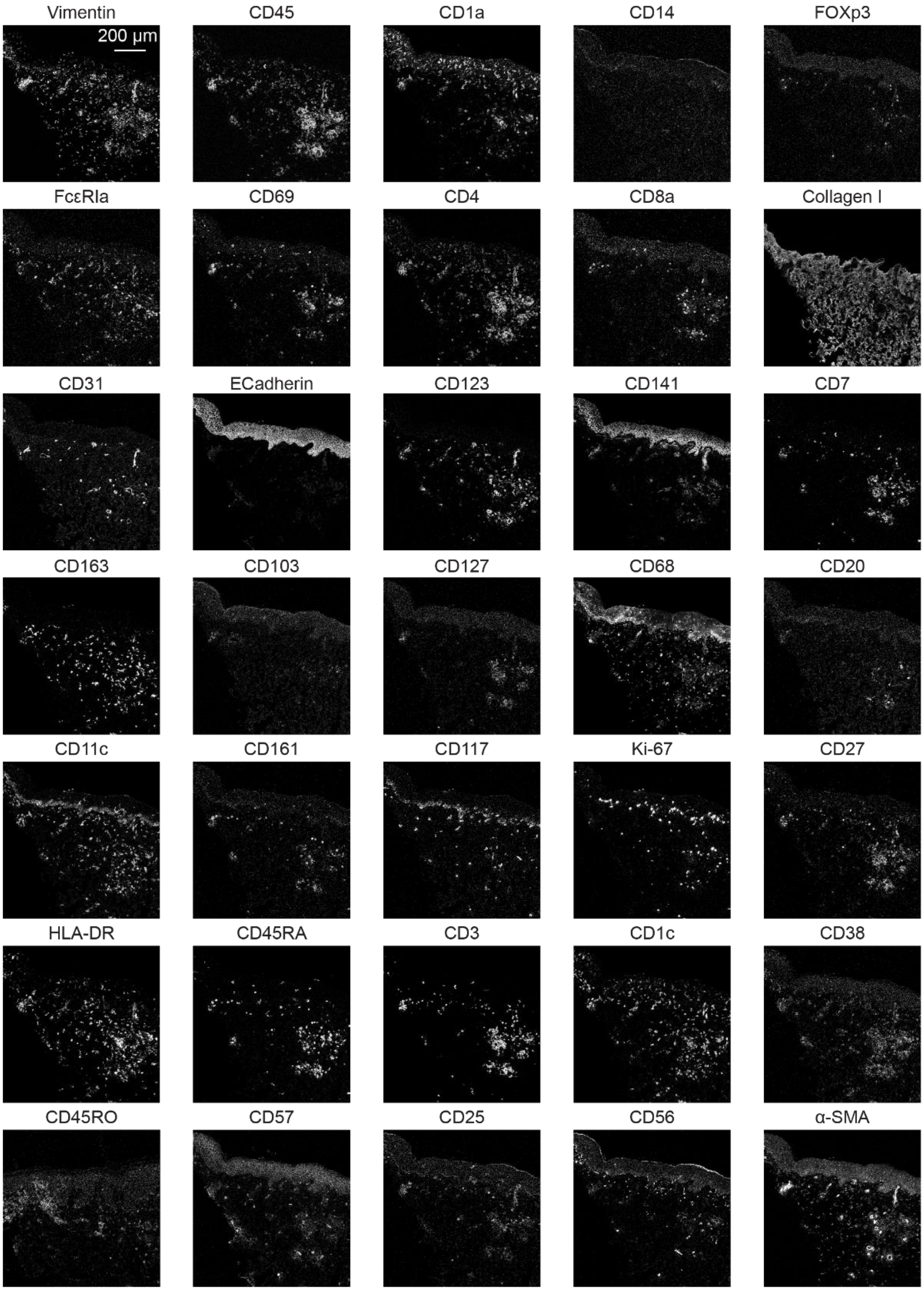

Figure S11 Individual antibody stains for 01NS donor by IMC.

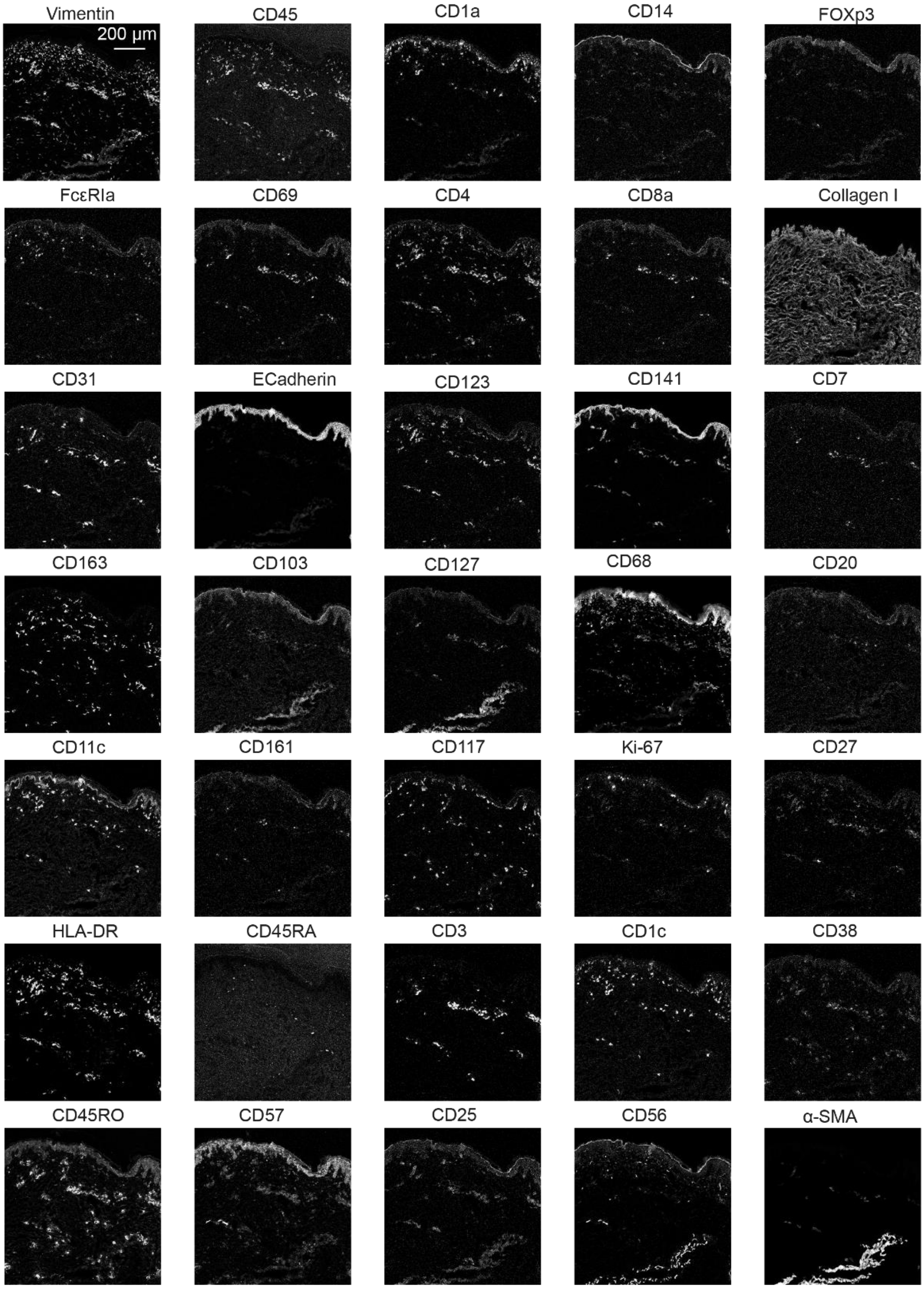

Figure S12 Individual antibody stains for 02NS donor by IMC.

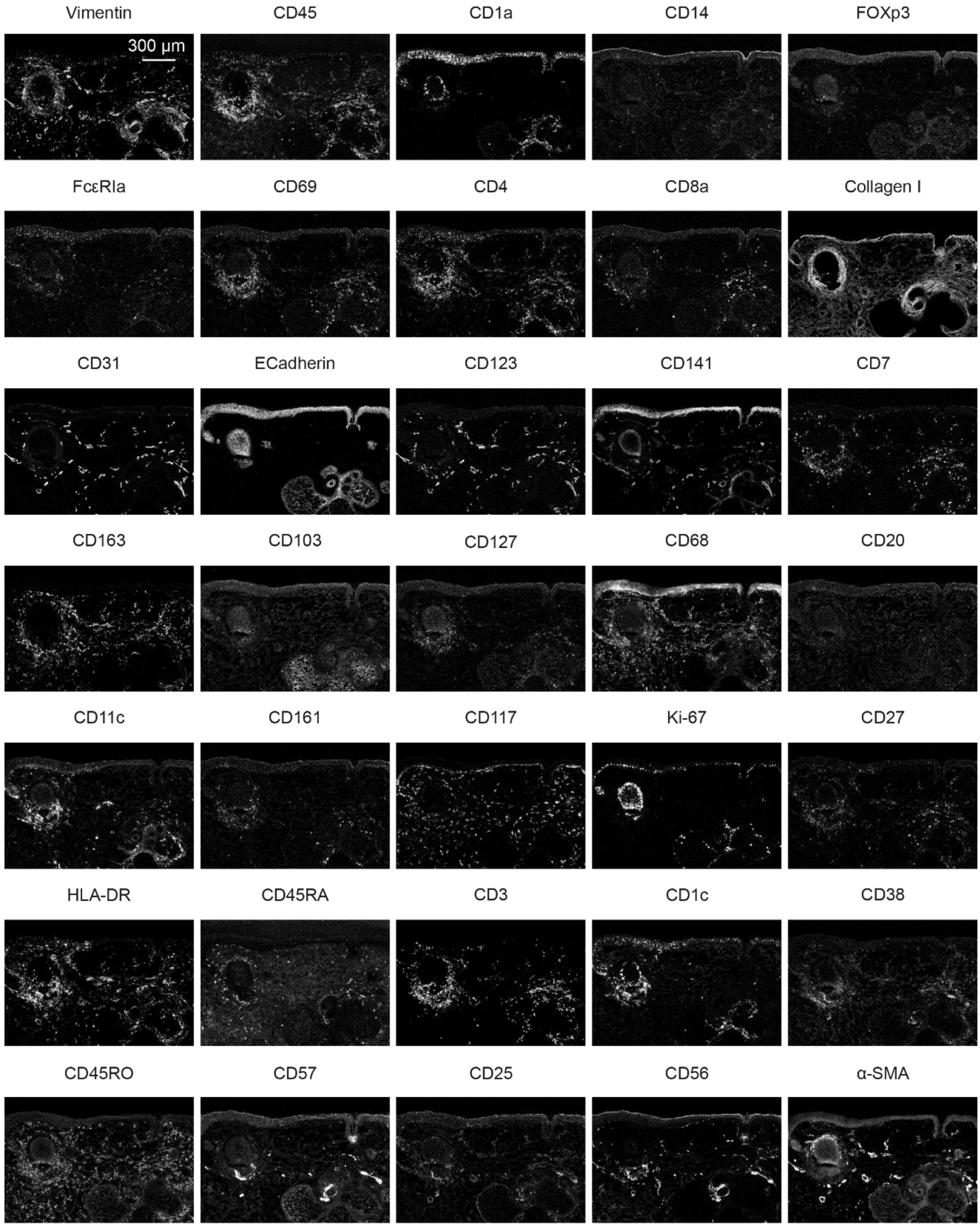

Figure S13 Individual antibody stains for 03NS donor by IMC.

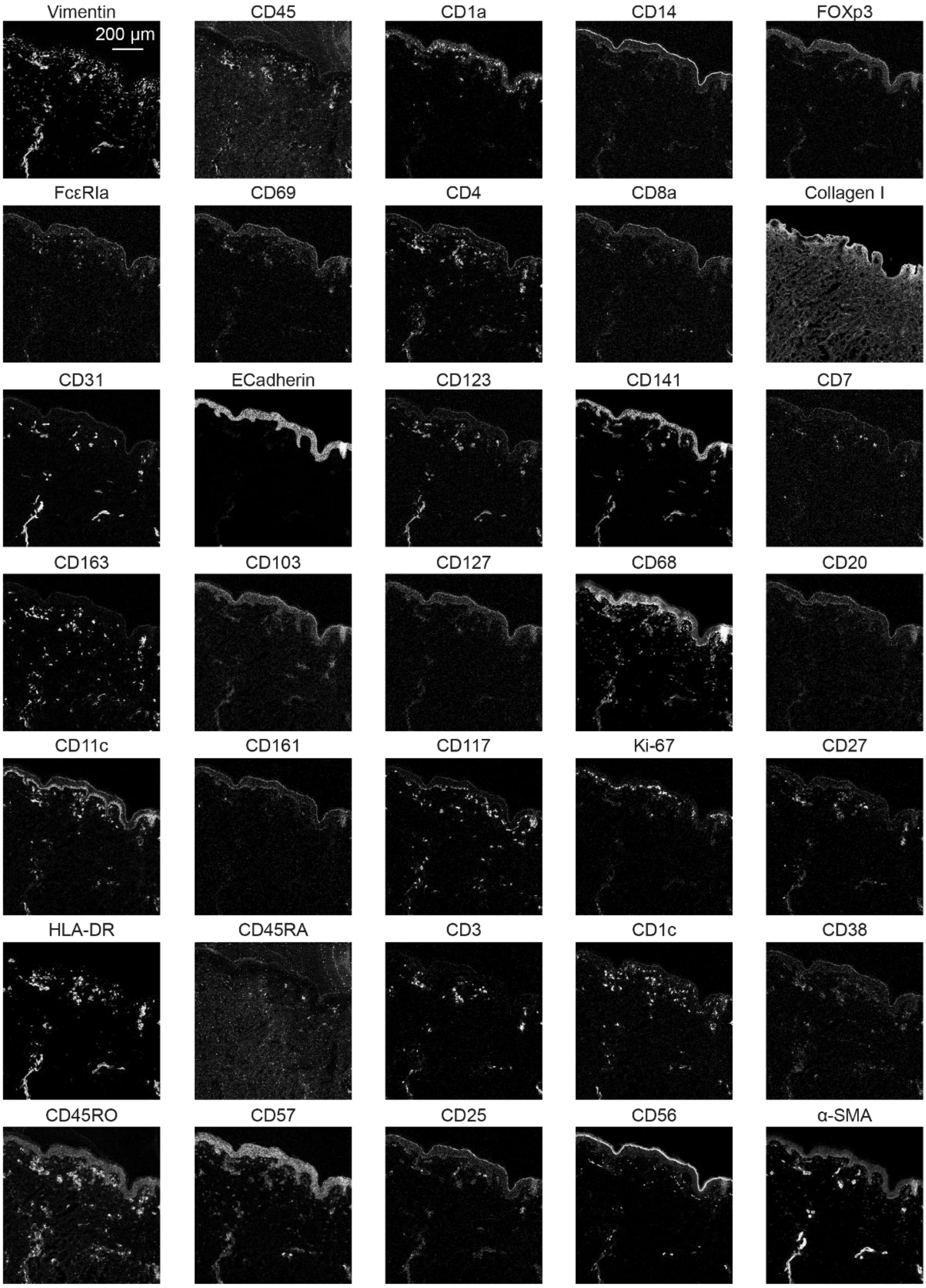

Figure S14 Individual antibody stains for 05NS donor by IMC.

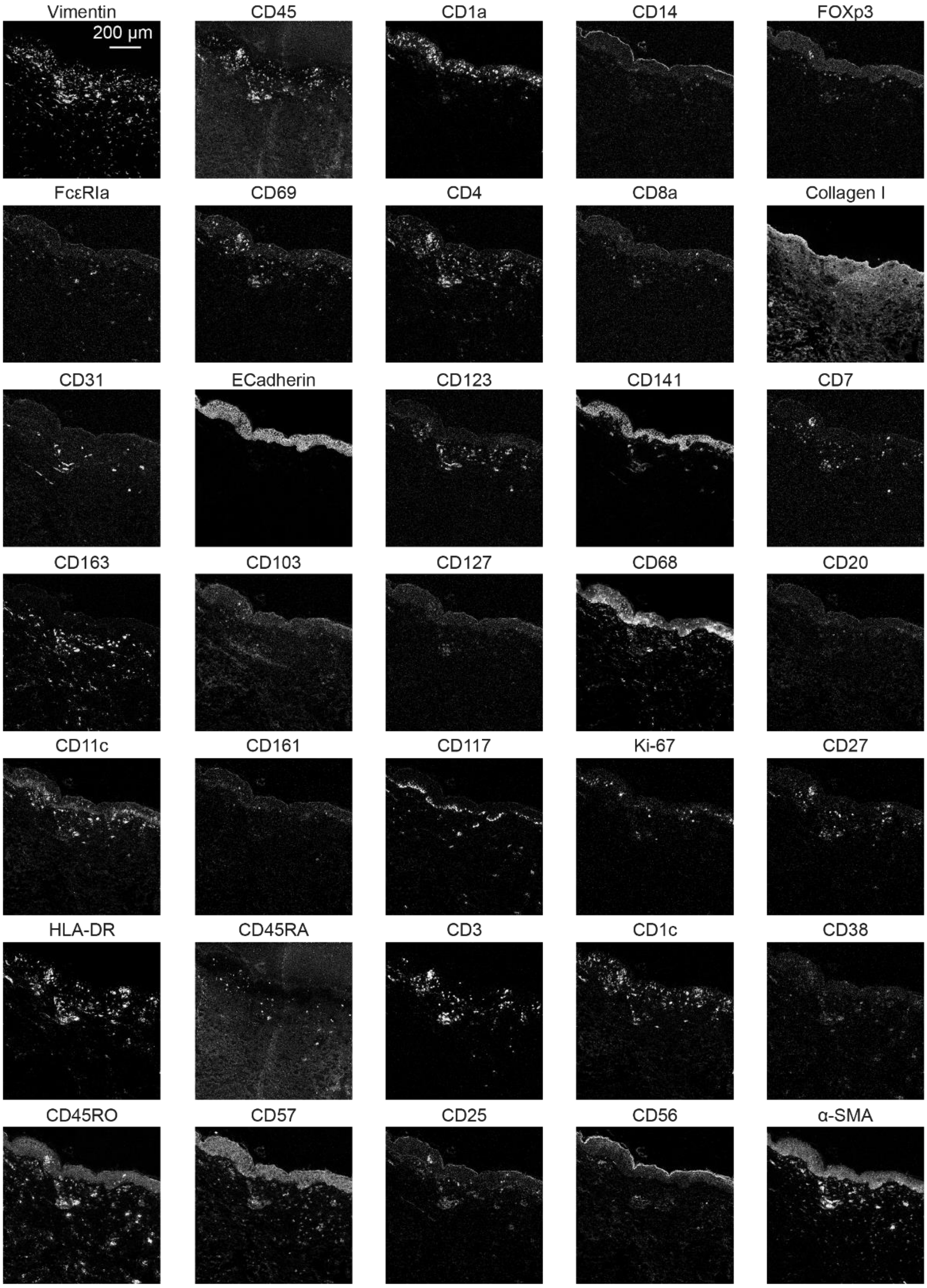

Table S1 Characteristics of patients with Mycosis fungoides diseases.

**Characteristics**	**MF patients by CyTOF (n=10)**	**MF Patients by IMC (n=6)**
Sex (male:female)	6:4	4:2
Years of MF prior to biopsy		
Median	2	4
Range	0-21	2-8
Age at biopsy, years		
Median	54.5	62.5
Range	34-76	41-76
Stage at biopsy		
IA	0	1
IB	10	4
IIB	-	1
Follow-up, months		
Median	15	12
Range	12-20	8-20
Therapy after biopsy		
Topical corticosteroids	10	6
UV-therapy	5	-
Retinoids	2	-
Interferon-alpha	1	-
Radiotherapy	1	1
Disease course		
PD	1	0
SD	5	5
PR	2	1
Unknown	1	

PD progressive Disease, SD stable disease, PR partial remission

Table S2 The sex and age characteristics of the NS healthy donors.

Heathy Donors	IMC/CyTOF	Sex	Age
NS01	IMC	Female	78
NS02	IMC	Male	76
NS03	IMC	Male	41
NS04	IMC	Male	77
NS05	CyTOF	Male	62
NS06	CyTOF	Male	34
NS07	CyTOF	Male	78
NS08	CyTOF	Female	68
NS09	CyTOF	Male	52
NS10	CyTOF	Male	62
NS11	CyTOF	Female	73
NS12	CyTOF	Male	70
NS13	CyTOF	Male	70
NS14	CyTOF	Male	42
NS15	CyTOF	Male	70
NS16	CyTOF	Female	87
NS17	CyTOF	Male	74
NS18	CyTOF	Female	92
NS19	CyTOF	Male	86
NS20	CyTOF	Male	41
NS21	CyTOF	Male	81

Table S3 Single-cell suspension mass cytometry antibody panel.

	Antigen	Tag	Clone	Supplier	Cat.	Dilution
1	CLA	104Pd	HECA-452	Biolegend	321302	1/50
2	CD39	108Pd	A1	Biolegend	328221	1/50
3	CD15	115In	W6D3	Biolegend	323035	1/50
4	CCR6	141Pr	G034E3	Fluidigm	31411003A	1/100
5	CD1a	142Nd	HI149	Sony	21100510	1/50
6	CD117	143Nd	104D2	Fluidigm	3143001B	1/100
7	CD69	144Nd	FN50	Fluidigm	3144018B	1/100
8	CD4	145Nd	RPA-T4	Fluidigm	3145001B	1/100
9	CD8a	146Nd	RPA-T8	Fluidigm	3146001B	1/200
10	CD57	147Sm	HNK-1	Biolegend	359602	1/200
11	CD16	148Nd	3G8	Fluidigm	3148004B	1/100
12	CD25	149Sm	2A3	Fluidigm	3149010B	1/100
13	IgM	150Nd	MHM88	Biolegend	314527	1/100
14	CD123	151Eu	6H6	Fluidigm	31511001B	1/100
15	TCRγδ	152Sm	11F2	Fluidigm	31512008B	1/50
16	CD7	153Eu	CD7-6B7	Fluidigm	3153014B	1/100
17	CD163	154Sm	GHI/61	Fluidigm	3154007B	1/100
18	CD103	155Gd	Ber-ACT8	Biolegend	350202	1/100
19	CRTH2	156Gd	BM16	Biolegend	350102	1/100
20	CD26	157Gd	BA5b	Biolegend	302702	1/100
21	CD30	158Gd	Ber-H2	Dako	M075101-2	1/50
22	CCR7	159Tb	G043H7	Fluidigm	3159003A	1/100
23	CD5	160Gd	UCHT2	Biolegend	300627	1/50
24	KLRG-1	161Dy	REA261	MACS	120-014-229	1/50
25	CD11c	162Dy	Bu15	Fluidigm	31612005B	1/200
26	CD20	163Dy	2H7	Biolegend	302343	1/200
27	CD161	164Dy	HP-3G10	Fluidigm	3164009B	1/100
28	CD127	165Ho	AO19D5	Fluidigm	3165008B	1/200
29	CD8b	166Er	SIDI8BEE	Ebio	15257407	1/50
30	CD27	167Er	O323	Fluidigm	3167002B	1/100
31	HLA-DR	168Er	L243	Biolegend	307651	1/300
32	CD45RA	169Tm	HI100	Fluidigm	3169008B	1/100
33	CD3	170Er	UCHT1	Fluidigm	3170001B	1/100
34	CD28	171Yb	CD28.2	Biolegend	302937	1/100

35	CD38	172Yb	HIT2	Fluidigm	31712007B	1/200
36	CD45RO	173Yb	UCHL1	Biolegend	304239	1/100
37	NKp46	174Yb	9E2	Biolegend	331902	1/40
38	PD-1	175Lu	EH 12.2H7	Fluidigm	3175008B	1/100
39	CD56	176Yb	NCAM16.2	Fluidigm	3176008B	1/100
40	CCR4	198Pt	205410	R & D	MAB1567-100	1/100
41	CD11b	209Bi	ICRF44	Fluidigm	3209003B	1/100
42	CD45	89Y	HI30	Fluidigm	3089003B	1/100
43	CD14	Qdot800	TüK4	ThermoFisher	Q10064	1/1000

Table S4 Imaging mass cytometry antibody panel on frozen skin tissue.

	Antigen	Tag	Clone	Supplier	Cat.	Dilution
1	CD1a	115In	010	Dako	M357101-2	1/50
2	CD14	141Pr	M5E2	BioL	301801	1/50
3	FOXp3	142Nd	236A/E7	eBioscience™	14-4777-82	1/100
4	FcεRIα	143Nd	AER-37 (CRA-1)	BioL	334602	1/50
5	CD69	144Nd	FN50	FLM	3144018B	1/50
6	CD4	145Nd	RPA-T4	FLM	3145001B	1/50
7	CD8a	146Nd	RPA-T8	FLM	3146001B	1/50
8	Collagen I	147Sm	polyclonal	Millipore	AB758	1/100
9	CD31	149Sm	8 9C2	CST	CST3528BF	1/100
10	ECadherin	150Nd	24 E 10	CST	CST3195BF	1/50
11	CD123	151Eu	6H6	FLM	3151001B	1/50
12	CD141	152Sm	Phx-01	BioL	902102	1/50
13	CD7	153Eu	CD7-6B7	FLM	3153014B	1/100
14	CD163	154Sm	GHI/61	FLM	3154007B	1/100
15	CD103	155Gd	EPR4166	Abcam	ab221210	1/50
16	CD127	156Gd	R34.34	Beckman	18LIQ494	1/50
17	CD68	159Tb	KP1	FLM	3159035D	1/200
18	CD20	161Dy	H1	FLM	3161029D	1/50
19	CD11c	162Dy	S-HCL-3	BioL	125602	1/50
20	CD11c	162Dy	Bu15	FLM	3162005B	1/50
21	CD161	164Dy	HP-3G10	FLM	3164009B	1/50
22	CD117	165Ho	104D2	BioL	313202	1/50
23	Ki-67	166Er	D3B5	CST	CST9129BF	1/200
24	CD27	167Er	O323	FLM	3167002B	1/50
25	HLA-DR	168Er	L243	BioL	307651	1/800
26	CD45RA	169Tm	HI100	FLM	3169008B	1/100
27	CD3	170Er	UCHT1	FLM	3170001B	1/100
28	CD1c	171Yb	L161	BioL	331501	1/50
29	CD38	172Yb	HIT2	FLM	3172007B	1/100
30	CD45RO	173Yb	UCHL1	BioL	304239	1/50
31	CD57	174Yb	HNK-1/Leu-7	Abcam	Ab212403	1/100
32	CD25	175Lu	24204.0	Thermo	MA5-23714	1/50
33	CD56	176Yb	NCAM16.2	FLM	3176008B	1/50
34	αSMA	194Pt	1A4	CST	56856BF	1/100

35	Vimentin	198Pt	D21H3	CST	5741BF	1/100
36	CD45	89Y	HI30	FLM	3089003B	1/50
Fluidigm (Flui), Cell Signaling Technology (CST) and Biolegend (BioL)						

Table S5. The minimum and maximum threshold of each marker for per sample.

		NS01		NS02		NS03		NS04		87MF		105MF		106MF		109MF		113MF		120MF	
Channel	Marker	Min	Max	Min	Max	Min	Max	Min	Max	Min	Max	Min	Max	Min	Max	Min	Max	Min	Max	Min	Max
Y(89)	CD45	3	7.56	3	12.69	3	6.87	3	6.54	2	17.24	1	13.42	1	14.08	1	14.63	1	9.14	1	14.95
In(115)	CD1a	1	9.78	1	17.14	1	8.64	1	14.85	1	13.54	1	14.23	1	10.38	1	7.84	1	9.52	1	6.9
Pr(141)	CD14	1	4.22	1	4.13	1	5.2	1	3.5	1	3.23	0.5	3.8	1	3.51	1	3.34	1	2.83	1	3.03
Nd(142)	FOXP3	1	3.51	1	3.94	1	3.68	1	3.42	1	4.13	1	3.39	1	3.17	1	3.3	1	2.95	1.5	4.73
Nd(143)	FceRIα	1	3.64	1	3.52	1	3.17	1	3.06	1	4.64	1	5.73	1	4.61	1	3.48	1	5.2	1	3.59
Nd(144)	CD69	1	5.96	1	6.42	1	4.19	1	5.83	1	9.07	1	7.86	1	6.69	1	12.43	1	9	1	9.13
Nd(145)	CD4	1	8.12	1	9.3	1	6.28	1	6.87	1	8.82	1	11.11	1	8.22	1	10.87	1	7.79	1	7.61
Nd(146)	CD8a	1	5.12	1	4.98	1	3.33	1	3.1	1	10.82	1	10	1	10	1	10	1	8	1	10
Sm(147)	Collagen I	3	22.54	3	40.83	3	18.44	3	16.54	3	36.48	3	28.47	3	35.67	3	44.56	3	37.37	3	41.06
Sm(149)	CD31	3	23.58	3	29.34	3	22.96	1	5.42	3	27.81	3	17.62	3	23.52	3	34.06	3	14.84	3	23.49
Nd(150)	ECadherin	3	44.14	3	55.93	3	37.57	3	32.67	3	27.2	3	28.28	3	30.02	3	28.08	3	26.68	3	24.93
Eu(151)	CD123	1	5.91	1.5	7.73	1	4.48	1	4.27	1	7.1	1	6.22	1	7.21	1	8.45	1	9.98	1	8.55
Sm(152)	CD141	1	22.26	3	27.95	3	17.13	3	16.54	3	15.22	3	21.35	3	17.98	3	19.68	2	15.44	3	13.94
Eu(153)	CD7	1	3.01	1	7.06	1	3.05	1	3.97	1	7.54	1	7.12	1	10.54	1	15.26	1	9.24	1	12.03
Sm(154)	CD163	3	30.43	1	20.83	1	16.89	2	15.73	2	54.73	3	32.38	3	36.23	2	41.86	1	20.47	1	23.33
Gd(155)	CD103	1	4.51	3	6.25	1	3.96	1	3.87	1	4.18	1	3.72	1	3.55	1	3.73	1	3.4	1	3.61
Gd(156)	CD127	1	6.4	1	4.11	1	3.29	0.5	3.13	1	4.58	1	3.84	1	3.8	1	4.75	1	3.51	1	3.68
Tb(159)	CD68	3	73.69	3	89.96	3	77.5	3	52.22	8	68.11	8	101.4	8	101.3	5	81.17	8	52.32	10	63.97
Dy(161)	CD20	1	3.44	2	3.63	1	3.4	1	3.14	1	3.47	1	3.34	1	3.16	1	3.2	1	3.64	1	3.23
Dy(162)	CD11c	3	9.55	3	13.65	3	8.46	3	7.06	3		3	14.13	3	12.33	3	14.89	2	13.34	3	16.88
Dy(164)	CD161	1	3.61	1	4.35	1	3.36	1	3.27	1	4.22	1	4.99	1	3.59	1	4.59	1	5.24	1	4.73
Ho(165)	CD117	1	13.62	1	13.62	1	11.85	1	15.73	1	15.46	1	10.99	1	13.61	1	9.14	1	9.16	1	6.33
Er(166)	Ki-67	1	8.35	1	19.62	1	13.21	1	5.79	1	13.6	1	25.18	1	21.68	1.5	25.96	1	15.77	1.5	16.44
Er(167)	CD27	1	4.55	0	5.45	1	6.43	1	7.31	1	10.76	1	7.14	1	6.5	1	7.57	1	6.67	1	6.73
Er(168)	HLA-DR	3	44.68	3	33.07	3	39.48	1	28.32	2	31.61	3	62.1	3	36.67	1	38.63	2	29.65	3	45.24

Tm(169)	CD45RA	3	8.84	3	8.64	3	9.55	3	7.76	3	70.32	2	21.36	2	20.26	3	21.42	2	25.68	2	25.35
Er(170)	CD3	1	25.74	1	20.68	1	16.74	1	17.71	1	29.95	1	20.8	1	25.46	1	27.53	1	18.94	1	22.41
Yb(171)	CD1c	1	10.98	1	10.54	1	9.62	1	9.47	1	13.22	1	17.29	1	12.87	1	9.8	1	13.53	1	16.51
Yb(172)	CD38	1	3.8	1	4.94	1	3.64	0.6	3.41	1	4.03	1	3.92	1	4.07	1	3.74	1	3.74	1	5.15
Yb(173)	CD45RO	3	28.42	3	51.66	3	21.86	3	20.36	3	26.56	3	26.05	3	23.51	3	27.58	3	23.54	3	26.98
Yb(174)	CD57	3	18.04	3	26.96	3	16.73	3	13.9	3	7.48	3	7.03	3	6.03	3	8.22	2	5.29	3	7.41
Lu(175)	CD25	1	4.17	1	4.35	1	4.32	1	4.24	1	5.21	1	4.32	1	3.97	1	3.99	0.5	3.67	1	6.28
Yb(176)	CD56	3	43.53	3	24.62	3	18.92	1	7.57	3	8.92	3	9.86	3	7.72	3	21.11	3	17.75	2	9.28
Pt(194)	αSMA	10	463.4	10	26.8	10	50.12	10	10.75	10	76.44	10	41.03	10	45.27	10	192.7	5	11.89	10	35.51
Pt(198)	Vimentin	5	129.4	5	175.8	5	117.4	5	95.46	5	175.5	5	113.5	5	117.7	5	116.3	3	116.5	5	117.8
